# Health disparities in one of the world’s most progressive countries: a scoping review of mental health and substance use among sexual and gender minority people in the Netherlands

**DOI:** 10.1186/s12889-023-17466-x

**Published:** 2023-12-18

**Authors:** Tonda L. Hughes, Lauren Bochicchio, Laurie Drabble, Maaike Muntinga, Jan S. Jukema, Cindy B. Veldhuis, Sunčica Bruck, Henny Bos

**Affiliations:** 1 School of Nursing, Department of Psychiatry, Columbia University Irving Medical School, Center for Sexual and Gender Minority Health Research, 560 West 168 Street, New York, NY USA; 2 Columbia University School of Nursing Center for Sexual and Gender Minority Health Research, 560 West 168th Street, New York, NY USA; 3 College of Health and Human Sciences, San Jose State University, One Washington Square, San Jose, CA USA; 4 Department of Ethics, Law and Humanities, Amsterdam UMC location VUmc, De Boelelaan 1118, Amsterdam, 1081 HZ Netherlands; 5 School of Health, Saxion University of Applied Sciences, M. H. Tromplaan 28, Enschede, 7513 AB Netherlands; 6 Departments of Medical Social Sciences, Psychology, Psychiatry, and Obstetrics & Gynecology, Northwestern University, 625 N. Michigan Ave., 14th Floor, Chicago, IL USA; 7 Present Address: Kohnstamm Instituut, Keizer Karelplein 1, Amstelveen, 1185 HL Netherlands; 8 Research Institute Child Development and Education, Faculty of Social and Behavioural Sciences, University of Amsterdam, Amsterdam, 1012 WX Netherlands

**Keywords:** The Netherlands, Dutch, Sexual and gender minority, SGM health, LGBTQ health, Minority stress, Structural stigma, Health disparities, Mental health, Substance use

## Abstract

**Background:**

Evidence from many parts of the world shows that sexual and gender minority (SGM) people have poorer health than their cisgender heterosexual counterparts. Minority stressors, particularly stigma and discrimination, have been identified as major contributors to sexual orientation- and gender identity-related health disparities, particularly negative mental health and behavioral health outcomes. To better understand factors that contribute to these disparities, we conducted a scoping review of SGM mental health and substance use research in the Netherlands—a country with a long-standing reputation as a pioneer in SGM equality.

**Methods:**

Using Joanna Briggs Institute guidelines and the PRISMA-ScR protocol, we searched seven databases to identify studies published between 2010 and 2022 that focused on substance use and/or mental health of SGM youth and adults in the Netherlands.

**Results:**

Although there was some evidence that SGM people in the Netherlands report fewer substance use and mental health concerns than those in less progressive countries, with very few exceptions studies found poorer outcomes among SGM participants than cisgender, heterosexual participants. However, this observation must be considered cautiously given major gaps in the literature. For example, only one study focused exclusively on adult sexual minority women, two focused on older SGM adults, and very little attention was given to nonbinary individuals. Most studies used non-probability samples that were quite homogenous. Many studies, especially those with youth, assessed sexual orientation based on sexual attraction; some studies of adults operationalized SGM status as having a same-sex partner. Importantly, we found no studies that directly assessed associations between structural-level stigma and health outcomes. Studies were mostly focused at the individual level and on health problems; very little attention was given to strengths or resilience.

**Conclusions:**

Findings of persistent health disparities—despite the relatively long history of SGM supportive policies in the Netherlands—highlight the need for more research and greater attention to population groups that have been underrepresented. Such research would not only provide guidance on strategies to improve the health of SGM people in the Netherlands, but also in other countries that are seeking to reduce health inequities. Addressing SGM health disparities in the Netherlands and elsewhere is complex and requires a multifaceted approach that addresses individual, interpersonal and structural factors.

According to the World Health Organization (WHO) “the enjoyment of the highest attainable standard of health is one of the fundamental rights of every human being without distinction of race, religion, political belief, economic or social condition” [1]. Further, the adoption of the United Nations 2030 agenda for sustainable development and its pledge to “leave no one behind” [2], based on the normative framework of international human rights law, reinforces the need to understand and improve the health and wellbeing of sexual and gender minority (SGM) populations. SGM populations include, but are not limited to, individuals who identify as lesbian, gay, bisexual, asexual, transgender, queer, and/or intersex, as well as those with same-sex or -gender attractions or behaviors [3].

Growing evidence from many parts of the world shows that compared to their cisgender heterosexual counterparts, SGM people have substantially poorer health [4–8]. Even in countries that have high levels of inclusive policies and more progressive attitudes toward SGM people, health outcomes appear to be worse than those of heterosexual people [9, 10]. Research on SGM health over the past few decades has consistently documented SGM-related health disparities, particularly in the areas of substance use and mental health. For example, SGM people are at disproportionately higher risk of harmful alcohol use and other substance use [11–14]; mental health concerns such as anxiety, depression and other forms of psychological distress [15–18], and suicidal thoughts and behaviors [19–26].

## Minority stress

In research across the globe, the most widely cited explanation for sexual and gender identity-related mental health disparities is minority stress [27, 28]. This theoretical perspective has expanded over time from its focus on sexual minority individuals to include gender minorities [29, 30], with a focus on the role of gender non-affirmation as a stressor for transgender and nonbinary people [31]. The minority stress model holds that prejudice, stigma, and cis/heteronormativity (the assumption that everyone is by nature heterosexual, and that everyone’s gender aligns with their birth-assigned sex) contribute to disparities via several primary mechanisms: (1) external, objective stressful events (e.g., discrimination, harassment, violence); (2) the expectation of such events and the vigilance that requires; (3) internalization of negative societal attitudes; and (4) rejection sensitivity, which often results in concealment of SGM status.

A particularly potent form of minority stress is structural stigma [9, 32, 33], defined as “societal-level conditions, cultural norms, and institutional policies that constrain opportunities, resources, well-being, and health of the stigmatized” [34] (p. 742). For example, Pachankis and colleagues [35] conducted a study using data from the 2017/18 European Men Who Have Sex with Men Internet Survey (N = 123,428), which assessed mental health and psychosocial mediators (sexual orientation concealment, internalized homonegativity, social isolation). These researchers linked data with an objective indicator of structural stigma related to sexual orientation (15 laws and policies and social attitudes). Among MSM who still lived in their country of birth, higher structural stigma was related to depression and suicidality via internalized homonegativity and social isolation. Among those who moved from higher-to-lower structural stigma countries, longer exposure to the lower structural stigma environments of their receiving countries was associated with lower risk of depression and suicidality as well as lower odds of concealment, internalized homonegativity, and social isolation. Further, studies of policies restricting same-sex marriage in the United States (U.S.) and Australia provide strong evidence of the negative impact of structural stigma on SGM people’s mental health [36–40].

## The Dutch context

To better understand how societal conditions, policies, and cultural norms impact of the health of SGM people, we conducted a scoping review of research related to the health of SGM people in the Netherlands. When considering social equality and acceptance of SGM people, the Netherlands is an interesting and somewhat complex case. It was trailblazer of anti-discrimination laws and the first country in the world to legalize same-sex marriage in 2001. These and its many other policies and laws that are supportive of SGM individuals and families place the Netherlands among the most SGM-friendly countries in the world. However, the Netherlands no longer leads in SGM equality. The International Lesbian, Gay, Bisexual, Trans and Intersex Association’s Europe Rainbow Index, most recently ranked it as 14th in Europe on measures of human rights and equality [41]. This compares to its ranking of 3rd in 2010 [42]. Nonetheless, other reputable sources continue to rank the Netherlands among the leaders in SGM equality. For example, based on the Global Acceptance Index which assesses acceptance of SGM individuals in 175 countries, the Netherlands is one of the most accepting countries in the world; in 2020, it was ranked, along with Canada, Iceland, Norway and Sweden, as one of the top five countries most accepting of SGM people [43]. Further, studies comparing the impacts of structural stigma in 28 countries European countries (see for example Pachankis, Hatzenbuehler [44], Bränström, Fellman [45], Bränström and Pachankis [46] report that the Netherlands has among the lowest levels of structural stigma based on an index score comprising measures of supportive and discriminatory laws and policies as well as country-level attitudes towards SGM people. These findings suggest that better understanding of the health and wellbeing of SGM people living in the Netherlands may help advance knowledge of factors that contribute to SGM health inequities.

Because a preliminary search found relatively few studies on the physical health of SGM Dutch people, and because evidence on the links between minority stress and substance use and mental health is much stronger [47], the following question guided this search: “What is known about mental health and substance use among SGM people in the Netherlands and how do findings compare with those of their cisgender, heterosexual counterparts?”.

## Methods

### Protocol and eligibility criteria

This study followed Joanna Briggs Institute guidelines for conducting scoping reviews [48]. We used the Preferred Reporting Items for Systematic Reviews and Meta-Analyses Extension for Scoping Reviews (PRISMA-ScR) protocol to report eligibility for study inclusion. Study inclusion criteria included empirical (both quantitative and qualitative) peer-reviewed articles published in English or Dutch from 2010 to 2022 that reported findings related to substance use and/or mental health among SGM individuals in the Netherlands. Studies including (presumably) heterosexual or cisgender individuals in the broader population were included if separate analyses of SGM people ages 12 and older were reported. Studies were excluded if they focused on individuals outside the SGM umbrella, or if they included children under age 12 and did not separately report outcomes for children older than 12.

### Search strategy and study selection

In June 2022, we searched seven databases to identify relevant studies. These included PubMed (pubmed.gov), PsycInfo (EBSCO), CINAHL (EBSCO), Embase (embase.com), Scopus (scopus.com), Gender Studies Database (EBSCO), and GenderWatch (ProQuest). Search terms were entered into the advanced search field in each database. The search resulted in 15,548 records. Duplicate records (n = 855) were removed in the citation manager, EndNote (version X9), prior to exporting the remaining 14,693 studies into Covidence, an online production tool, for a second duplicate record removal, title/abstract screening, full-text screening, and data abstraction.

**Fig. 1:**
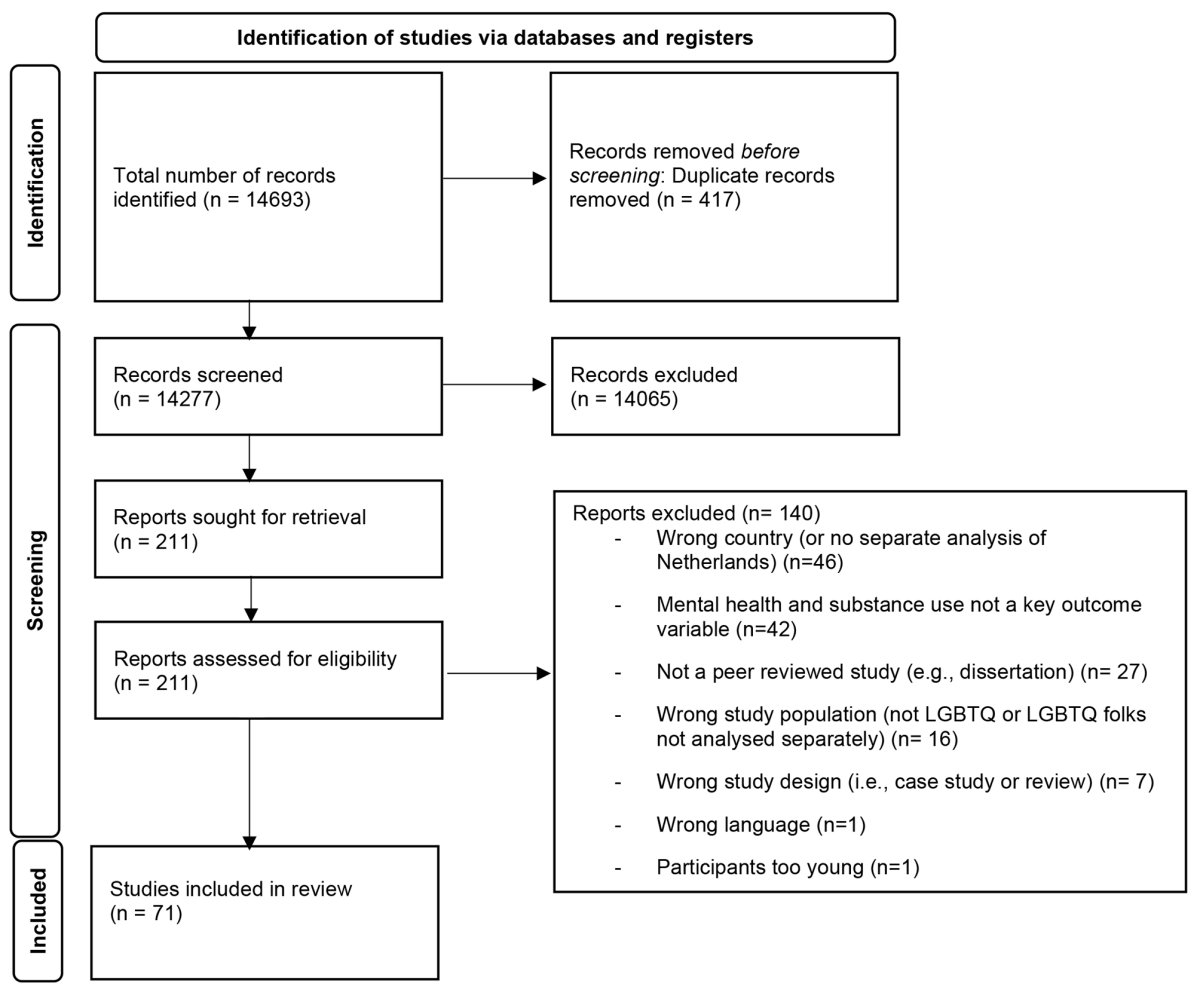
Prisma-ScR Diagram

Once exported into Covidence, 412 additional duplicate records were identified and removed. This resulted in 14,281 unique records that were screened for possible inclusion. All authors were involved in the screening process. At least two authors independently screened the title and abstract of each article and removed those that did not meet inclusion criteria. A total of 14,065 articles were removed during this stage, leaving 211. Two authors reviewed each full-text article. At this stage, 140 studies were excluded for one or more of the following reasons: wrong country (or no separate analysis of data from the Netherlands), wrong outcome (mental health or substance use were not a key outcome), not peer-reviewed (e.g., dissertations), wrong study population (not SGM or SGM participants were not analyzed separately), wrong study design (i.e., case study or review), wrong age group (i.e., participants were younger than age 12), or wrong language (article not written in Dutch or English). When discrepancies between authors arose at any stage, we consulted a third author to resolve the disagreement through a consensus-based process. A total of 71 studies were included in the final review. See Prisma flowchart in Fig. 1 for summary of the screening process [49].

### Data extraction, analysis, and synthesis

We extracted key study data from each of the 71 studies into a data extraction table. Although all authors independently contributed to data extraction, the first and second authors separately reviewed 25–30% of articles and extracted data as a quality check of consistency and accuracy of that data. The following categories were included in the data extraction table: study location, language (Dutch or English), study design, sample size, participant demographics, dependent variable(s), independent variable(s), and main outcomes. Table 1 provides a summary of key characteristics of the 71 studies. Data were then assessed on the aggregate level and are presented here using a descriptive synthesis of results. Given important developmental differences and differences in health concerns we chose to present results separately for youth (children/adolescents) and for adults. Like most research that includes findings for sexual minorities and gender minorities separately, we separated these two population groups; this is particularly important given differences in some key health concerns of the two groups. Summarizing results in this manner is intended to facilitate understanding of available evidence and identification of gaps in the literature.

**Table 1:** Sample Characteristics and Study Design

Author (Year)	Type of Data/ Study design	Outcomes	Study Location	Total sample size (N)	Sample	Heterosexual comparison group	Sexual Identity	Age of sample (M, Mdn, Range)
Achterbergh et al. (2020a)	Primary data, cross-sectional, quantitative	Mental health/ Substance use	Netherlands	4,461	MSM	No	Men only sexual partners: Amsterdam: 92% Surrounding Amsterdam: 80% Both men and women sexual partners: Amsterdam: 8% Surrounding Amsterdam: 20%	Mdn: 35
Achterbergh et al. (2020b)	Primary data, prospective cohort study	Substance Use	Amsterdam, Netherlands	341	Trans women, gay, bisexual, and other MSM	No	All participants reported same-sex partners	Mdn: 40
Achterbergh et al. (2020c)	Secondary analysis, cross sectional	Substance Use	Netherlands	1,130	MSM	No	All participants reported same-sex partners	Mdn: 40
Achterbergh et al. (2021)	Primary, RCT, longitudinal	Mental health/ Substance use	Amsterdam, Netherlands	115	MSM	No	All participants reported same-sex partners	Intervention: Mdn: 46 Control: Mdn: 42
Aggarwal & Gerrets (2014)	Primary, qualitative (ethnographic)	Mental health	Amsterdam, Netherlands	12 MSM, 10 experts, and stakeholder consultations	MSM	No	All MSM identified as gay	Range: 25–70
Alberse et al. (2019)	Primary, cross-sectional, quantitative	Mental health	Amsterdam, Netherlands	674	TGD youth	No	Not included	Children: 6–13 Adolescents: 11–18
Arnoldussen et al. (2020)	Primary, longitudinal, quantitative	Mental health	Amsterdam, Netherlands	1,072	TGD youth	No	Not included	Range: 10.1–18.1
Asscheman (2011)	Other: clinical reports, cohort study	Mental health	Netherlands	1,331	TGD youth and adults	No	Not included	MTF Range: 16–76 FTM Range: 16–56
Baams et al. (2013)	Primary, cross-sectional, quantitative	Mental health	Netherlands	192	SM youth	No	SMM: 45%, SMW: 55%	Range: 16–24
Baams et al. (2014)	Primary, cross-sectional, quantitative	Mental health	Amsterdam, Netherlands	325	SSA youth	No	Not included	Range: 16–24
Baams et al. (2018)	Secondary, cross-sectional, quantitative	Mental health	Netherlands	267	SSA youth	No	Gay: 19%, lesbian: 30%, bisexual: 22%, queer: 2%, other: 6%, no response: 20%	Range 16–22
Baams et al. (2021)	Secondary, cross-sectional, quantitative	Mental health	Netherlands	6,393	SSA adults	Yes	Exclusively other SA: 91%, predominantly other SA: 7%, both sex attracted: 0.83%, predominantly SSA: 0.48%, exclusively SSA: 1%	M: 44.2
Biggs (2020)	Clinical reports, Longitudinal	Mental health	Amsterdam, Netherlands	70	TGD youth	No	Not included	M: 14.8
Bos (2010)	Primary, cross-sectional, quantitative	Mental health	Netherlands	72	Gay fathers	Yes	Gay men: 50%	Gay M: 44.08, Heterosexual: M: 39.78
Bos et al. (2014)	Primary, cross-sectional, quantitative	Mental health	Netherlands	1,546	SM adolescents	Other: Yes	Non-SSA boys: 49%, non-SSA girls: 51%, SSA boys: 42%, SSA girls: 58%	Non-SSA group: M: 14, SSA group: M: 14
Bos et al. (2015)	Secondary data from NESDA, cross-sectional, quantitative	Mental health/ Substance use	Netherlands	1,780	LGB	Yes	SSA: 7%, heterosexual: 93%	Heterosexual men: M: 43; SMM: M: 45, Heterosexual women: M 40, SMW: M: 40
Bos et al. (2016)	Primary, cross-sectional, quantitative	Substance use	Netherlands	703	SSA adolescents	Yes	SSA: 10.1%	Range: 14–20
Cense et al. (2017)	Primary, mixed method, quant	Mental health	Netherlands	Quant: 576, Qual: 18	transgender	No	N/A	Quant study: Range: 18–76 Qual study: not provided
Collier et al. (2013)	Secondary, cross-sectional, quantitative	Mental health	Netherlands	513	LGB	Yes	SSA: 11.1%	Range: 11–17
Coyer et al. (2022)	Primary, prospective cohort study	Substance use	Amsterdam, Netherlands	976	HIV-negative MSM	No	100% MSM, 79% identified as exclusively homosexual	Mdn: 33.2
de Graaf et al. (2018)	Cross-sectional, comparative	Mental health	Amsterdam, Ghent, London, Zurich	959 total	TGD adolescents referred to gender identity clinics	No	None reported	NLD: M: 14, UK: M: 15.5, Belgium: M: 14, Swiss: M: 15
de Graaf & Picavet (2018)	Primary, cross-sectional, quantitative	Mental health	Netherlands	3,054	SSA Adults	No	14% had a same-sex partner, 25% had an opposite-sex partner	M: 46.58, range: 14.35
de Graaf et al. (2021)	Primary, cross-sectional, quantitative	Mental health	Gender identity clinics at university hospitals in Amsterdam and United Kingdom	Study 1: 589 Study 2: 632	Study 1: clinic referred gender diverse adolescents Study 2: clinic referred gender diverse adults	No	Not reported	Study 1: AMAB: M:16, AFAB: M:16 Study 2: Range 17–67
de Graaf et al. (2022)	Primary, cross-sectional, quantitative	Mental health	Toronto, Amsterdam, and London	2,771	Adolescents presenting with gender dysphoria	Other: No cisgender comparison group	focus was on gender identity; sexual orientation not assessed	M: 16
de Neve-Enthoven et al. (2016)	Primary, national follow-up audit, quantitative	Mental health	Nijmegen, Rotterdam, Amsterdam, Netherlands	120	Persons with a DSD diagnosis	No	Not reported	Range: 14–60
de Vries et al. (2011)	Longitudinal observational descriptive cohort study	Mental health	Amsterdam, Netherlands	70	Gender dysphoric adolescents	No	SSA: 89%, Sexually attracted to both sexes: 9%, Other: 3%	Range: 11–17; M 14
de Vries et al. (2016)	Primary, cross-sectional, quantitative	Mental Health	Toronto, Canada, and Amsterdam, Netherlands	316	Gender dysphoric adolescents	No	Not reported	Amsterdam: Range: 13–18, Toronto: Range: 13–18
Drückler et al. (2018)	Secondary, cross-sectional, quantitative	Substance use	Amsterdam, Netherlands	4,925	MSM	Yes	MSM 31%, Non-MSM 69%	Range: 27–46
Drückler et al. (2020)	Secondary analysis of electronic patient files, Cross-sectional survey at STI clinic	Substance use	Amsterdam, Netherlands	99	TSW, MSW-M, MSW-F	Other: Compared male sex workers and trans women sex workers	TSW: 15%, MSW, M:70%, MSW-F:15%	Range: 25–35
Evers et al. (2020)	Primary, cross-sectional, quantitative	Substance use	Netherlands (public STI clinics)	511	MSM	No	100% MSM	Range: 27–50
Feddes & Jonas (2020)	Primary, cross-sectional, quantitative	Mental health	Netherlands	391	SGM adults	No	Lesbian: 23%, gay: 61.1%, bisexual: 7.4%, Not specified: 8.4%	M: 49.4
Gevonden et al. (2014)	Secondary, cross-sectional from two time periods, quantitative	Mental health/ Substance use	Netherlands	Study 1 from 1996: 5,927 Study 2 from 2007-9: 5,300	SM adults (only assessed attraction/ behavior)	Yes	Heterosexual: study 1: 98%, study 2: 97.8%; LGB: study 1: 2%, study 2: 2.2%	Heterosexual: study 1 M:41; study 2 M:39; LGB (study 1 M:43.5; study 2 M:42
Ghassabian et al. (2022)	The study was embedded in Generation R, longitudinal	Mental health	Rotterdam, Netherlands	5,727	TGD Youth	Other: cisgender comparison group	Not reported	Parents and their children, ages 9–11 and/or 13–15 years
Heiligenberg et al. (2012)	Primary, cross-sectional (lab data were used for STI outcomes)	Substance use	Amsterdam, Netherlands	2,822	MSM	Yes	MSM: 24% Heterosexual men: 34% Heterosexual women: 42%	Range: 23–37
Heylens et al. (2014)	Primary, cross-sectional, quantitative	Mental health	Amsterdam, Netherlands; Ghent, Belgium; Hamburg, Germany and Oslo, Norway	305	Adults with gender identity disorder seeking gender reassignment therapy and surgery	None	Not reported	Netherlands: FTM: M: 31 MTF: M: 36 All Countries: FTM: M: 28.5, MTF: M: 35
Jafary & Ashrafi (2022)	Primary, cross-sectional, quantitative	Mental health	Iran, Netherlands	124	Gay men	No	All participants identified as gay	Range 20–50; M 33
Kaufman et al. (2017)	Primary, cross-sectional, quantitative	Mental health	Netherlands	364	LGBT youth	No	Lesbian 30%, Gay-19%, Bisexual- 22%, Queer: 2%, Other sexual identity: 6%, Did not answer: 21%	Range: 16–22 years; M 18
Kaufman et al. (2020)	Secondary, longitudinal, quantitative	Mental health	Netherlands	2,222	LGB youth	Yes	LGB: 7%	W1: M: 11, W2: M: 14, W3: M: 16, W4: M: 19, W5: M: 22
Kiekens et al. (2020)	Secondary, longitudinal, quantitative	Mental health/ Substance use	Netherlands	1,738	LGB	Yes	LGB: 7%	W1 M: 11, W2 M: 14, W3: M: 16, W4: M: 19, W5: M: 22
Kiekens et al. (2022)	Primary, longitudinal, quantitative	Substance use	Netherlands	393	SGM Youth	No	Lesbian/gay: 44%. Bisexual:30%, Queer: 7%, Pansexual: 10%, Heterosexual: 1%, Don’t know: 5%, Other SM: 3%	16–25; M:18.36; 57% younger than age 18 (legal drinking age)
Kuyper & Fokkema (2010)	Primary, cross-sectional, quantitative	Mental health	Netherlands	161	LGBs	No	LGB 78%, Somewhat over 40% of the participants were women.	Range: 55–85; M 65
Kuyper & Fokkema (2011)	Secondary, cross-sectional, quantitative	Mental health	Netherlands	396	LGB Adults	No	Gay men; 30% bisexual men: 10% lesbian women: 47% bisexual women: 13%	Range: 10–70
Kuyper (2014)	Secondary, cross-sectional, quantitative	Mental health	Netherlands	8,064	TGD Individuals	No	Not Reported	Range: 15–70
Kuyper (2015)	Secondary, cross-sectional, quantitative	Mental health	Netherlands	9,417	LGB Adults	Yes	Heterosexual: 94%, Bisexual: 3%, Gay/lesbian: 3%	41.54 years
Kuyper (2016a)	Secondary, cross-sectional, quantitative	Mental health	Netherlands	5,995	SSA youth	Yes	SSA: 2% Not yet attracted (NYA): 6%, Heterosexual: 92%	SSA M: 14; NYA M: 12 Heterosexual M: 13
Kuyper (2016b)	Secondary, cross-sectional, quantitative	Mental health/ Substance use	Netherlands	4,403	SSA and same-sex partnered adults	No	Same-sex partnered: community sample: 88%, panel sample: 71%	Panel sample: M 48 Community sample: M 37
Kuyper & Bos (2016)	Primary, cross-sectional, quant	Mental health/ Substance use	Netherlands	528	LGB Young Adults	No	Mostly heterosexual: 81%, lesbian/gay: 19%	Range: 16–25; M: 21
la Roi et al. (2016)	Secondary, longitudinal cohort, quantitative	Mental health	Netherlands	1,738	LGB Youth	Yes	Heterosexual boys: 45%, lesbian girls: 49%, Lesbian/gay: 2%, Bisexual girls/boys: 6%	Range: 11–22
Leyerapf et al. (2018)	Primary, cross-sectional, quantitative	Mental health	Two major cities, Netherlands	64	LGBT older adults and care professionals	No	Did not provide exact information	14 LGBT and 16 heterosexual participants, 55+
Motmans et al. 2012	Primary, cross-sectional, quantitative	Mental health	Gender identity clinic at a university hospital, Netherlands	225	Trans people	No	Not Reported	M: 40
Nikkelen & Kreukels (2018)	Primary, cross-sectional, quantitative	Mental health	Netherlands	576	Trans people	No	Other: No information about sexual identity	Range: 17–76; M: 42
Parra et al. (2021)	Primary, cross-sectional, quantitative	Mental health	Netherlands	675	LGB	No	SSA: 7%, Both-sex attraction: 23.5%;	Range: 18–29: M: 22
Sandfort et al. (2010)	Secondary cross-sectional, quantitative	Mental health	Netherlands	513	SM adolescents	Yes	SSA: 11%	Range 12–15; M: 14
Sandfort et al. (2014)	Primary, cross-sectional, quantitative	Mental health	Netherlands	6,646	SSA adults	Yes	Men with same-sex behavior 2%, Women with same-sex behavior 2%, SSA Men: 2.5%, SSA Women: 2.5%	M: 44
Schouten et al. (2011)	Primary, cross-sectional, quantitative	Mental health	Netherlands	57	Men and women with homosexual feelings/ behaviors	Yes	SSA/same sex behaviors: Islamic: 5%, Dutch indigenous: 5%	M: 26
Schrijvers et al. (2020)	Primary, cross-sectional, quantitative	Mental health	Netherlands fertility clinic	95	SMW in relationships	Yes	Women in same-sex relationship: 26%	M: 33
Shirdel-Havar et al. (2019)	Primary, cross-sectional, quantitative	Mental health	Amsterdam, Netherlands, and Poona, Iran	163	Trans men and trans women	Other: N/A	Netherlands: SSA: 61.5% both sexes: 10%, other sex: 27%, Iran: SSA: 67% both sexes: 21%, other sex: 16%	Netherlands: M: 32 Iran M: 25
Steensma et al. (2011)	Primary, qualitative	Mental health	Amsterdam, Netherlands	53	Gender dysphoric children/adolescents	No	Not Reported	Baseline: M: 9, Follow-up: M: 16; Range:14–18
Steensma et al. (2013)	Clinical reports, longitudinal, quantitative	Mental health	Amsterdam, Netherlands	127	Dutch adolescents referred and diagnosed in childhood with gender dysphoria	No	Attraction: Boys: 19% attracted to other sex, 15% attracted to both sexes, and SSA 65%, Girls: 32% attracted to the same sex, 5% attracted to both sexes, 63% SSA	Range: 7–19
Steensma et al. (2014)	Primary, cross-sectional, quantitative	Mental health	Canada and Netherlands	728	TGD children and adolescents	No	Not reported, all participants met criteria for gender identity disorder or gender identity disorder not otherwise specified	Amsterdam: M: 10.0 Toronto: M: 9.0
Stoffelen et al. (2018)	Primary, qualitative	Mental health	Netherlands	10	SMW with mild ID	No	100% Lesbian or bisexual	Range: 20–49
Tornello et al. (2018)	Secondary, cross-sectional, quantitative	Mental health	Netherlands	5,857	People in same sex relationships	Yes	In a same sex relationship: 3%	Range:18–79
Van Bergen et al. (2013)	Secondary cross-sectional, quantitative	Mental health	Netherlands	274	LGB	No	SSA Girls, 61%, SSA Boys 39%	M: 17
Van Bergen & Spiegel (2014)	Primary, qualitative	Mental health	Netherlands	30	LGB	No	SSA Girls: 50% and SSA Boys: 50%	Range 16–25
Van Beusekom et al. (2016)	Primary, cross-sectional, quantitative	Mental health	Netherlands	1,026	SSA youth	Yes	No SSA: 92%, Rarely SSA:4%, Sometimes SSA: 2%, Frequently SSA: 1%, Very often: 0.6%	Range: 11–16; M:13
Van Beusekom et al. (2018)	Primary, cross-sectional, quantitative	Mental health	Netherlands	724	LGB	No	Male: 93% gay, 7% bisexual, Female: 73% lesbian, 27% bisexual	Range: 18–73; M: 32
Van de Grift et al. (2017)	Clinical reports, longitudinal	Mental health	Amsterdam, Netherlands	21	Transgender men	No	Female partner: 63%, Male partner: 10.5%	M: 40
Van den Akker et al. (2013)	Secondary, cross-sectional, quantitative	Mental health	9 European Countries	82,797	Not reported	Yes	Not reported	Not reported
Van der Miesen et al. (2020)	Clinical data, cross-sectional, quantitative	Mental health	Gender identity clinic at a university hospital in Amsterdam, Netherlands	272	TGD adolescents starting the assessment process and TGD adolescents receiving affirming care	Other: Cisgender comparison group	Not reported	Adolescents starting the assessment process: M: 14.5 years; TGD adolescents receiving affirming care: M:17 years; General population adolescents: M: 15 years
Verbeek et al. (2020)	Primary, qualitative	Mental health	North Netherlands	20	TGD adults who completed transition	No	Not reported	Transgender men M:43, Transgender women M:8.5; Range: 20–75
Wiepjes et al. (2020)	Primary, clinical records	Mental health	Amsterdam, Netherlands	8,263	TGD Youth	No	Not reported	Mdn age at first visit: 25

Disorders of Sex Development (DSD), Intellectual Disability (ID), Female to Male (FTM), Lesbian, Gay, Bisexual (LGB), Lesbian, Gay, Bisexual, Transgender (LGBT), Male to Female (MTF), Men who Have Sex with Men (MSM), Male Sex Workers who Have Sex with Females only (MSW-F), Male Sex Workers who Have Sex with Men and Women (MSW-M), Mean (M), Median (Mdn), Sexual and Gender Minority (SGM), Sexual Minority (SM), Sexual Minority Men (SMM), Sexual Minority Women (SMW), Same-sex Attracted (SSA), Transgender and Gender Diverse (TGD), Transgender Women Sex Workers (TSW)

## Results

### Mental health - sexual and gender minority youth

#### Sexual minority youth (SMY)

Fourteen studies examined mental health among SMY. Seven of these focused on psychological distress and wellbeing [50–56], five on mental health outcomes such as anxiety and depressive symptoms [16, 57–60], and two focused on suicidality. Only one study used qualitative methods [55].

Of the 14 studies, two used data from the TRacking Adolescents’ Individual Lives Survey (TRAILS), a population-based cohort study of youth who were followed from early adolescence (age 10–12 in 2000) into young adulthood [16, 60]. The other studies collected primary data, and all drew on the minority stress model for their theoretical framework, solely or in combination with other theoretical perspectives (e.g., interpersonal-psychological theory, psychological mediation).

#### Studies comparing SMY and heterosexual youth

Five studies investigated factors contributing to differences in psychological wellbeing between sexual minority youth/emerging adults and their heterosexual counterparts [16, 50–52, 60]. Overall, these studies found that SMY reported greater psychological distress than heterosexual youth. For example, disparities in mental health outcomes were documented in the two longitudinal studies using data from the TRAILS study [16, 60]. Kaufman, Baams [60], drawing on five waves of data from both adolescent and parent reports, found that lesbian, gay and bisexual (LGB) adolescents were more likely than their heterosexual peers to be victimized and to be victimized over a long period of time. LGB adolescents who were persistently victimized reported higher levels of internalizing problems, mainly anxiety, than heterosexual youth. la Roi, Kretschmer [16] examined the developmental period in which disparities in depressive symptoms between heterosexual and LGB youth start to occur, how these disparities develop over time, and factors contributing to them. LGB youth showed an overall higher risk of depressive symptoms than heterosexual youth. Discrepancies between LGB and heterosexual youth were larger for LB girls than for GB boys, and for bisexual than gay and lesbian youth, perhaps because most bisexual-identified study participants were girls. In addition, the development of depressive symptoms followed a different pattern for boys and for girls. By age 11, LB girls were at higher risk of depressive symptoms than heterosexual girls, and these differences increased over time. Sexual identity differences in depressive symptoms were partially mediated by peer victimization and parental rejection.

Several researchers recruited samples from schools. Bos, van Beusekom [50] recruited participants (N = 1,546) from 12 secondary schools in the Netherlands. They found that the relationships between same-sex attraction (SSA) and low self-esteem, and between SSA and psychological distress, were partially mediated by passive coping style. This mediation effect was similar for male and female youth [50]. In a study of students in eight Dutch secondary schools, Sandfort, Bos [51] found that students with and without SSA did not differ in regard to their psychological distress in schools that had consistent and clear rules and expectations about behavior toward others. The school context appears to be an important factor in mental health and wellbeing among SMY. Using the same dataset as Bos, van Beusekom [50], researchers found that nearly half (47.2%) of all student participants reported having been called homophobic names by at least one person, such as a classmate, in the past month; male adolescents and SSA adolescents reported this more often than female adolescents [52]. Each of these studies used same-sex attraction, rather than identity, as the indicator for sexual minority status and each found disparities in mental health outcomes among SSA youth.

#### Studies focusing on within group differences

Several studies examined correlates of mental health among SGM youth using cross-sectional designs. Baams [57] and Kaufman, Baams [59] investigated whether the feeling of being a burden to others (burdensomeness) [57] and rumination (repetitive thinking about negative feelings or events) [59] helped explain associations between sexual minority stressors and depressive symptoms. Both studies focused on sexual minority youth and emerging adults (16–22 years old). Baams [57] found that sexual orientation–based victimization and internalized homophobia were indirectly related to depressive symptoms through perceived burdensomeness, but not through thwarted belongingness. Furthermore, indirect associations between minority stress and depressive symptoms were not dependent on problem-solving coping. Kaufman, Baams [59] found that the association between microaggressions and depressive symptoms was mediated by rumination. Youth who experienced microaggressions were more likely to use ruminative emotion regulation in response, which was linked to higher levels of depressive symptoms. Sexual minority-specific support did not buffer the relationship between microaggressions and depressive symptoms. In another study, Baams [57] examined associations between sexual minority stressors and psychological wellbeing. Expected rejection, feeling that most heterosexual people have negative attitudes toward homosexuality (meta-stereotyping), and internalized homophobia were significant predictors of lower levels of wellbeing. The negative impact of expected rejection (but not other minority stressors) was buffered by being in a romantic relationship [53].

In a study of 106 female and 86 male same-sex attracted (SSA) youth (16–24 years old), Baams, Beek [54] found that participants with high levels of gender non-conformity reported higher levels of perceived stigmatization due to their sexual orientation, which was in turn associated with lower levels of wellbeing. These associations did not differ between male and female study participants [54]. van Beusekom, Baams [56] explored gender nonconformity, homophobic peer victimization and mental health among a sample of 1,026 Dutch adolescents aged 11–16 years. Mediation analyses revealed that homophobic name-calling mediated the associations between gender non-conformity and both social anxiety and psychological distress. Additionally, the researchers found a moderated mediation effect: the effect of mediation was greater when the level of SSA was higher.

Two studies, Parra, van Bergen [22] and Van Bergen, Bos [26], used cross-sectional designs to examine the association between victimization and suicidality among SMY. The prevalence of lifetime suicidal ideation was high in both studies (56.7% and 63.9%). Van Bergen, Bos [26] found that 12.8% of the sample had previously *attempted* suicide and the relationship between victimization and suicidality varied based on the context in which the victimization took place. For example, school-based victimization was significantly associated with suicidal ideation and suicide attempts; however, family-based victimization was associated only with suicide attempts, and neighborhood victimization was associated only with suicidal ideation. Parra, van Bergen [22] found that homophobic violence, entrapment (extent to which participants experience cognitions of feeling trapped in their lives and current situations or circumstances) and family belongingness were associated with suicidal ideation. Additionally, family support moderated the relationship between entrapment and suicidal ideation, such that the effect of entrapment on suicidal ideation was lower among sexual minority emerging adults who reported familial support.

In the only qualitative study of mental health among SMY, van Bergen and Spiegel [55] examined coping responses to stigma in a sample of 30 SSA youth (15 girls and 15 boys) ages 16–26 years. Study findings highlighted four distinct patterns of coping: avoidant coping, moving beyond avoidant coping (initially anxious and avoidant but moving toward healthier coping), healthy emotional coping, and direct problem-solving. Patterns of coping with stigma varied, as did the ability to critique “heteronormativity,” based on social resources and social networks (especially from family, peers, and LGB organizations), and personal strengths.

#### Transgender and gender diverse (TGD) youth

Fourteen studies addressed mental health of Dutch TGD youth: most focused on clinic-referred children/adolescents, and several compared clinical samples with non-clinical samples of cisgender peers, non-Dutch clinical samples, or both. Five studies included a comparison group from another country (i.e., Canada, the United Kingdom, Switzerland, Belgium). All except one of the studies used quantitative methods; Steensma, Biemond [61] conducted a qualitative study using biographical interviews to describe changes in gender dysphoria among adolescents.

In one of the few studies to draw on non-clinical samples, Ghassabian, Suleri [62] used data from Generation R, a Rotterdam population-based cohort of children born between 2002 and 2006 (N = 5727). Mental health was assessed at ages 13–15 years. Youth with gender-variant experiences (defined as wishing to be the opposite sex and/or to be treated as someone of the opposite sex) were more likely to report adverse mental health outcomes, such as anxiety and depression. Ghassabian, Suleri [62] found that parent reports of their child’s gender variant experiences increased as children aged. For children ages 9–11, 1% of parents reported gender-variant experiences, and this number increased to 4% among children who were 13–15 years. The authors also found that adolescents assigned female at birth (AFAB) reported more gender-variant experiences than those assigned male at birth (AMAB).

#### Gender affirming treatment and psychological wellbeing

Several studies examined the impact of gender-affirming medical treatment on psychological wellbeing. De Vries, Steensma [63] compared functioning of 70 TGD adolescents with gender dysphoria, before and after starting puberty blocking medication. Although participants’ reports of gender dysphoria did not change after the start of puberty suppression, there were significant reductions in reports of behavioral and emotional problems and symptoms of depression. Further, although general functioning improved significantly, there were no significant changes in overall reports of anxiety or anger. Biggs [64] compared the Dutch sample used in the De Vries, Steensma [63] study with a UK sample of TGD adolescents, and the improvement in psychological functioning after treatment observed in the Dutch sample was not replicated in the UK sample. van der Miesen, Steensma [65] compared clinic-referred adolescents who received puberty suppression with two groups: clinic-referred adolescents who had not yet started this treatment and cisgender peers. The pre-treatment group reported a higher number of internalizing problems, self-harm and suicidal ideation, and poorer peer relations than their non-referred peers. In contrast, adolescents who had started puberty suppression had fewer emotional and behavioral problems than adolescents who had not yet started treatment, and similar or fewer problems than their cisgender peers [65].

Studies that compared Dutch clinic-referred samples to similar samples outside the Netherlands generally found that TGD adolescents in the Netherlands experienced fewer mental health problems than TGD adolescents in other countries [64, 66–68]. For example, in a cross-national study of clinic-referred adolescents in the Netherlands, UK, Switzerland and Belgium, those from the Netherlands reported the lowest number of behavioral, emotional, and peer relationship problems [69]. In a study that compared suicidality across Dutch, UK and Canadian samples, de Graaf, Steensma [70] found that clinic-referred adolescents had higher rates of suicidality than the comparison samples. Adolescents from the Amsterdam clinic had lower suicidality scores than adolescents referred to the Toronto and London clinics, and AFAB adolescents were more likely to report suicidality [70].

Alberse, de Vries [71] found that children and adolescents referred to a transgender clinic in Amsterdam had lower self-perception related to their bodies and lower self-worth than a comparison group of cisgender (non-referred) children and adolescents. AFAB children and adolescents were more self-satisfied than AMAB peers, but only in childhood did they think themselves to be superior to non-referred peers in the domains of sport, school, and social acceptance. Steensma, McGuire [72] observed a link between the intensity of early dysphoria and its persistence over time. AFAB children and those who were older at initial assessment were more likely to report persistent gender dysphoria. Other predictors of persistent gender dysphoria included cognitive and/or affective cross-gender identification, as well as social role transition in childhood. These factors varied among AFAB and AMAB children. Reporting on the relationship between nonbinary identity and mental health outcomes among clinic-referred adolescents, de Graaf, Huisman [73] found that nonbinary identity was associated with psychological problems such as anxiety, agoraphobia, depression, interpersonal sensitivity, hostility, and sleep deprivation.

Arnoldussen, Steensma [74] examined trends in gender dysphoria and gender-affirming care among TGD adolescents referred to a clinic in Amsterdam between 2000 and 2016. They found no change in the percentage of clinic adolescents diagnosed with gender dysphoria (75–95% of all referrals) or in those receiving gender-affirming medical care (54–95%).

### Substance use – sexual and gender minority youth

Four studies examined substance use among SGM youth [75–78]. Overall, findings point to greater substance use among SMY than heterosexual youth, and findings varied based on sex/gender and how SGM status was operationalized. Bos, van Beusekom [76] compared any alcohol use, quantity of alcohol consumed, and drinking motives in SMY and heterosexual youth 14 to 20 years old. SMY were more likely than heterosexual youth to drink alcohol on weekdays, and to use alcohol to cope with worries and to conform to group social norms. Using alcohol to cope with worries mediated the relationship between sexual orientation and drinking during the week and was stronger for boys than for girls. Kiekens, Baams [75] used a daily diary method to examine alcohol use and minority stress in a sample of 409 SGM youth (mean age 18.36). Youth completed a daily online survey about their alcohol use, experiences of prejudicial events, expectations of rejection, concealment of their identity, and experience of internalized homophobia. Findings showed few significant associations between minority stressors and alcohol use, but daily experiences of concealment and prejudice events were associated with daily alcohol use and these associations varied by sex assigned at birth and gender identity, respectively.

Two studies [78, 79] included substance use outcomes and psychological health measures, such as psychosomatic complaints, emotional problems, and internalizing problems. In a cross-sectional study using data from the HSCB study, Kuyper and Bos [79] examined differences between adolescents who reported SSA and those who did not (non-SSA), as well as those who reported not knowing who they felt attracted to (NYA). Compared to the non-SSA adolescents, those with SSA reported more frequent substance use (e.g., alcohol, tobacco, and cannabis), lower levels of life satisfaction, and higher levels of psychosomatic complaints and emotional problems. Findings from the NYA group were inconsistent.

In a longitudinal study using data from the first five waves of TRAILS, Kiekens, la Roi [78] examined the links between LGB identity and internalizing problems and substance use through a serial mediation process. They hypothesized LBG identity would be associated with peer victimization and negative relationships with parents, which, in turn, would be associated with fear of negative social evaluation and lack of social support. Those factors, then, would lead to increases in internalizing problems and substance use. LGB youth had higher scores on internalizing problems and reported more smoking and marijuana use than heterosexual youth. Unexpectedly, being victimized was associated with lower likelihood of substance use. The association between sexual identity and externalizing problems (e.g., substance use) was mediated by peer victimization and parental rejection [78].

### Mental health – sexual and gender minority adults

#### Sexual minority adults

Of the 19 studies that examined mental health among sexual minority adults, four focused exclusively on men who have sex with men (MSM) [80–83], two on sexual minority women (SMW) [84, 85], and 13 on LGB adults more broadly [66, 86–98]. Most studies found that compared to their heterosexual counterparts, LGB adults reported poorer mental health and quality of life.

Several studies using large general population samples documented disparities in mental health by sexual orientation. Sandfort, de Graaf [86] used data from the Netherlands Mental Health Survey and Incidence Study-2 (NEMESIS-2), a prospective study among Dutch-speaking subjects aged 18–64 years (N = 6646) from the general Dutch population, to examine associations between same-sex sexuality (i.e., same-sex attraction or behavior) and psychiatric disorders. Participants reporting same-sex sexuality were more likely than those reporting only opposite-sex sexuality to meet criteria for DSM-IV psychiatric disorders. Moreover, disparities in psychiatric disorder prevalence were greater in studies that compared same-sex and opposite-sex attraction than those comparing same-sex and opposite-sex behavior [86]. Gevonden, Selten [87] used cross-sectional data from NEMESIS-1 and NEMESIS-2 to examine the associations between sexual minority status and psychotic symptoms. LGB participants (defined as having sexual relations with at least one same-sex partner during the past year) were more likely to report childhood trauma, childhood bullying, past-year discrimination, and psychosis-related symptoms than their counterparts who reported only different-sex partner. Past year discrimination mediated 34% of the association between LGB status and psychosis-related symptoms, bullying mediated 7%, childhood trauma mediated 5%, substance use mediated 3%, and living without a partner mediated 11% [87]. Similarly, Baams, Ten Have [58] used data from NEMESIS-2 to explore adverse childhood experiences and DSM-IV disorders between same-sex attracted participants (reporting exclusive or predominant attraction to people of the same sex or attraction to both sexes) and exclusively other-sex attracted individuals. Same/both-sex attracted individuals were more likely than those with only other sex-attraction to report every type of childhood trauma and bullying victimization assessed, and more likely to report severe childhood trauma. Childhood trauma severity and bullying victimization partly explained differences in mental health for same/both-sex attracted individuals.

Two panel sample studies also identified disparities using different measures of sexual orientation. In a large (N = 3054) panel sample of adults who reported any same-gender attraction [66], participants who fit a solely same-gender attracted and early minority sexual identity trajectory, and who reported no different-gender sexual experiences, had higher levels of psychological wellbeing than those in the same-gender attracted, but different-gender sexual experiences trajectory. In the first wave of the Netherlands Kinship Panel Study (N = 5857), Tornello, Ivanova [89] found that participants in same-gender relationships reported somewhat lower life satisfaction than their peers in different-gender relationships, but were no differences in reports of partner support or couple conflict. Higher partner support was associated with higher life satisfaction and higher relationship conflict was associated with lower life satisfaction. However, whereas couple conflict was negatively associated with life satisfaction among different-gender couples, this was not the case among same-gender couples.

Only one study focused explicitly on mental health outcomes in relation to gender non-conformity. In a convenience sample of 724 LGB adults, van Beusekom, Baams [56] found that participants who reported higher levels of gender nonconformity showed overall poorer mental health than those who reported lower levels of gender nonconformity. Although internalized homophobia mediated the relationship between gender nonconformity and mental health for both men and women, homophobic stigmatization was a significant mediator among men only. Gender nonconformity was not significantly related to homophobic stigmatization for lesbian and bisexual women, suggesting differences in the level of stigmatization of gender nonconformity between men and women.

Two cross-cultural studies examined mental health outcomes by sexual identity. Jafary and Ashrafi [80] examined adult attachment and emotion regulation strategies (i.e., cognitive reappraisal and expressive suppression) among Iranian gay men (40 Iranians residing in Iran and 41 Iranians who had immigrated to the Netherlands) and 43 Dutch gay men. Compared to both groups of Iranian gay men, Dutch gay men reported more confidence in their relationships and were more likely to use cognitive reappraisal than emotional suppression to regulate their emotions. Iranian participants residing in the Netherlands reported higher levels of emotional suppression than Dutch participants, but lower levels than Iranian men living in Iran. Additionally, compared to Dutch men, gay Iranian immigrants in the Netherlands reported higher levels of anxiety in situations in which they felt rejected or used—but again, they had lower levels of anxiety than Iranian gay men living in Iran. In another cross-cultural study, Schouten, Knipscheer [92] compared the mental health of 57 Islamic immigrants and 61 indigenous Dutch homosexual [sic] identified adults in the Netherlands to 1,009 Dutch adults in the general population. Islamic and indigenous Dutch sexual minorities reported significantly more symptoms of anxiety and depression than heterosexual adults. Analyses comparing Islamic and indigenous sexual minority participants found no significant differences.

Only two mental health studies focused specifically on SMW. Stoffelen, Schaafsma [84] explored experiences of coming out, sexuality, mental health, and discrimination among 10 lesbian and bisexual women with mild intellectual disabilities. Most study participants reported insecurity, extreme loneliness, depression, alcohol addiction, and anxiety. Additionally, several participants reported experiences of bullying and discrimination across social contexts. Schrijvers, van Rooij [85] found that among women seeking fertility counseling (N = 95), those in lesbian relationships (n = 10) were most likely (40%) to report unmet needs. In general, women in the study had good mental health, but 14% met criteria for clinically significant mental health problems. Across groups, women with more unmet counselling needs also had higher levels of internalizing and externalizing problems than women without unmet needs.

In a five-wave prospective study (2002 to 2010; N = 82,797) that focused on the health of LGB people living in nine European countries characterized by high levels of support and favorable opinions of LGB people, including the Netherlands, van den Akker, Blaauw [90] found that compared to people in different-gender relationships, those in same-gender relationships reported poorer health and lower happiness, and this was particularly the case for those who also reported experiencing discrimination. LGB people who reported discrimination also reported significantly worse health and lower happiness than their LGB counterparts who reported no discrimination. Feddes and Jonas [91] examined associations among experiencing LGBT hate crimes, intentions to report future experiences of hate crimes, and psychological wellbeing in a sample of 319 LGB adults. 16% of participants reported having experienced a hate crime in the 12 months preceding the survey. Experiences of victimization and stigma among LGB individuals were associated with lower trust in police as well as lower intention to report future hate crimes. Psychological wellbeing partially mediated this relationship: individuals with higher psychological wellbeing had higher trust in police and greater intentions to report future hate crimes.

Some studies focused broadly on mental health in specific contexts such as work, school, and family. In a cross-sectional study of workplace experiences among 9,417 employees, Kuyper [94] found no differences between lesbian/gay and heterosexual participants on measures of bullying, unequal treatment, job satisfaction, and burnout. However, bisexual women reported higher levels of bullying, unequal treatment, and burnout than lesbian or heterosexual women. Similarly, bisexual men reported higher levels of burnout than gay and heterosexual men. An ethnographic study of 12 gay men in the Netherlands [81] found that gay men reported feeling the need to censure their identity and gender expression at work, with family, and when participating in team sports. Additionally, gay men reported struggling with feelings of loneliness and a degraded sense of self-worth from an early age due to experiences of rejection. Bos [83] studied experiences of fathers by sexual identity, finding no differences in emotional involvement, parental burden, or child wellbeing; however, gay fathers experienced more rejection and feelings that they had to defend their status as fathers.

In the only study that focused on a mental health intervention, Achterbergh, van Rooijen [82]) tested a syndemic based intervention with 115 MSM. The study assessed the effectiveness of providing screening for mental health-related problems and tailored feedback aimed at increasing help-seeking behavior and decreasing sexually transmitted infection (STI) risk among MSM. At baseline, almost all participants reported at least one mental health problem; 20% reported four mental health problems. Screening for mental health-related issues, providing tailored advice, and referrals to mental health and addiction treatment services did not increase help-seeking behavior among study participants.

Only two studies examined mental health among older LGBT adults. Leyerzapf, Visse [97] examined experiences and needs of LGBT older people concerning their inclusion and participation in care settings. The researchers used multi-stakeholder interviews, participant observation and focus groups in three elderly care homes in the Netherlands that had been recognized for their efforts in creating a gay-friendly climate. Despite this, participants in the study reported feeling categorized as “different” from other residents and feeling either socially invisible or hyper visible. They felt the need to stay secretive about their LGBT identity, partner status, and to try to pass as “normal” heterosexual people. Participants who were more open about their sexual identity reported experiencing discrimination (e.g., being called a “dyke”) and social exclusion. In a quantitative cross-sectional study, Kuyper and Fokkema [96] examined associations between loneliness and minority stress in a sample of 161 older LGB adults. The researchers found that loneliness was positively associated with experiences of prejudice and expectations of prejudice. Further, participants with fewer LGB social connections reported greater loneliness than those with a larger LGB social network. LGB older adults who reported negative reactions or discrimination also reported higher levels of loneliness than younger LGB people and those with stronger LGB social networks.

### Mental health - transgender and gender diverse (TGD) adults

Ten studies focused on mental health among TGD adults. These studies generally found that TGD adults suffered from poorer mental health and that gender affirmation treatment was associated with improvements in mental health and wellbeing. For example, Motmans, Meier [99] found that compared to men in the Dutch general population, transgender men had significantly lower quality of life. However, there were no differences between transgender women and Dutch women in the general population. In the qualitative portion of a study by Cense, de Haas [100], transgender participants reported experiencing PTSD, depression, dissociation, negative self-image, and low self-confidence.

Kuyper and Wijsen [101] explored gender identity and gender dysphoria in a Dutch general population sample (N = 8,064, ages 15–70 years old). Results were that 4.6% of AMAB participants and 3.2% of AFAB participants reported feeling bigender (i.e., both male and female) while 1.1% of the AMAB participants and 0.8% AFAB participants reported identifying with the opposite gender. Among participants who expressed a bigender or transgender identity, only 0.6% of AMAB participants and 0.2% reported disliking their natal body and/or wishing for hormones/surgery.

Heylens, Elaut [102] found that affective and anxiety disorders were more common in adults applying for gender affirmation treatment than among the general Dutch population; no differences were found between the two groups in terms of personality disorders or intellectual developmental disorders. In a qualitative study of 20 Dutch TGD adults in Northern Netherlands, Verbeek, Hommes [103] found that transgender individuals reported improved psychological wellbeing since transitioning, and findings emphasized the value of social and peer support in this regard. Similarly, both van de Grift, Pigot [104] and Nikkelen and Kreukels [105] found a positive association between sexual activity/feelings and use of genitals following completion of genital-gender confirmation surgery.

Two studies examined rates of suicide among TGD adults. Wiepjes, den Heijer [106] found that between 1972 and 2017, rates of completed suicide among transgender women decreased, whereas no change was observed in the rate of suicide among transgender men. Additionally, the average number of suicides between 2013 and 2017 was higher in the transgender sample than in the general population. Asscheman, Giltay [107] examined rates of suicidal behavior and other factors linked to premature mortality among transgender individuals. Compared to cisgender men in the general population, transgender women showed a 51% higher mortality rate. This difference was primarily attributed to higher rates of suicide, illicit drug use, and AIDS among transgender women. There was no significant difference in rates of mortality between transgender men and cisgender women.

Finally, in a cross-cultural study of transgender individuals in Iran and the Netherlands, Shirdel-Havar, Steensma [108] compared the two groups on several mental health indicators. Participants in Iran scored higher than Dutch participants on measures of most mental health disorders (e.g.,, anxiety, agoraphobia, depression, sleeping problems). In addition, transgender women in Iran reported significantly higher dissatisfaction with primary and secondary sex characteristics, whereas transgender women in Netherlands reported higher dissatisfaction with gender neutral characteristics (e.g., feet, nose). Irrespective of country of origin, transgender women scored significantly higher than transgender men on anxiety and agoraphobia.

### Intersex individuals

de Neve-Enthoven, Callens [109] investigated psychosocial wellbeing among 120 participants aged 14–60 years from three medical centers in the Netherlands who were diagnosed with disorders of sex development. They assigned study participants to one of three groups: (1) 46 XY and female genitalia, (2) 46 XY or 46 XX, and atypical genitalia, and (3) men with 46 XY and atypical genitalia). Data from the three groups were compared to data from Dutch patient groups with chronic conditions that impede independent daily functioning and self-care. Individuals with 46 XY reported good health-related quality of life, no serious emotional problems, a high self-esteem, and seemed to cope well compared to the Dutch reference groups [109].

### Substance use – sexual and gender minority (SGM) adults

#### Sexual minority adults

Nine studies focused on substance use among sexual minority adults. Seven of these collected data primarily or solely from MSM [110–117]; two of these studies included non-MSM comparison groups [115, 117]. Another study collected data from sex workers who were MSM, transgender, or men who had sex with women [114]. One study focused on comparisons of study participants who identified as lesbian or gay with those who identified as “mostly heterosexual” [79].

Most studies in this section used cross-sectional survey designs [79, 111, 113–117]; two used a prospective cohort study design [110, 112]. Most (n = 6) were conducted in Amsterdam [110, 112–115, 117]. One study collected data from multiple sexually transmitted infection (STI) clinics in the Netherlands [116], another from a commercial panel sample [79], and one combined secondary data from four different datasets [111].

All except one of the studies [79] focused on MSM who engage in chemsex (i.e., sexual activity while under the influence of drugs) [111, 115–117] and/or understanding the relationship between drug use and risk of HIV or STIs [110, 112–114]. In general, studies found high rates of chemsex among MSM, with even higher rates among HIV-positive MSM compared to HIV-negative and non-MSM populations [117], and among MSM who visited STI clinics compared to those who did not [115]. Coyer, Boyd [110] found that recreational drug use, specifically chemsex, increased over time in their prospective cohort study. MSM who were polydrug users reported more sexual partners than those who used no drugs, fewer drugs, or only used alcohol [111, 113]. A prospective study of mental health and drug use among MSM who use PrEP found lower rates of sexual compulsivity and drug use disorders, but no changes in mood disorders or alcohol use disorders over time [112]. Sex workers who identified as MSM or transgender had similarly high rates of using illicit drugs while engaged in sex work (approximately 40%), with “sex work becomes physically easier” as the most commonly reported reason for substance use [114]. Approximately 25% of MSM who participated in chemsex reported a need for counseling about chemsex-related issues [116]. A study of psychological distress, minority stress and substance use among mostly heterosexual and lesbian/gay individuals [79] found no differences in binge drinking but higher levels of drug use, smoking, psychological distress, and suicidality among mostly heterosexual participants compared to lesbian/gay participants. They also found that higher levels of internalized negativity related to SSA mediated the relationship between psychological distress and substance use. These researchers combined women and men in their analyses, making it difficult to understand possible sex/gender differences.

## Discussion

We reviewed available peer-reviewed research published between 2010 and 2022 that focused on mental health and/or substance use among SGM youth and adults in the Netherlands. There was some evidence that SGM people in the Netherlands report fewer substance use and mental health concerns than SGM people in other, less progressive countries—in line with the assumption that supportive polices and environments are important to the mental health and wellbeing of SGM individuals. At the same time, with very few exceptions, studies included in the review reported more mental health concerns and more substance use among SGM participants than among their cisgender, heterosexual counterparts.

SGM people in the Netherlands, particularly youth, gay men, and TGD people, reported experiencing minority stress in multiple contexts (e.g., school, work, older adult care settings); findings related to SMW and to bisexual men were too limited to draw conclusions. Evidence suggests that despite relatively low levels of structural stigma in the Netherlands, many Dutch SGM people feel the need to censor their identity and gender expression in many contexts. Findings from Dutch gay men in the Aggarwal and Gerrets [81] study and older Dutch SGM adults in the Leyerzapf, Visse [97] study indicate that such censoring is linked to experiences (or fear) of rejection. Social rejection and social isolation are powerful determinants of health outcomes [118], and older SGM adults are especially vulnerable to social isolation [119]. Findings such as these raise the question: Given the broad range of supportive policies aimed at protecting the rights of SGM people in the Netherlands, what factors are associated with persistent SGM-related health disparities?

Unfortunately, the results of this review shed limited light on this question. Compared to some other western countries, such as Australia, Canada, and the U.S., where research on SGM health is rapidly growing, Dutch research on SGM health is quite limited. It is possible that researchers and funders have not seen the need for such research, given that SGM people are perceived to be widely accepted in the country [120], whereas acceptance in the U.S. and in other countries that are major producers of SGM research is more mixed. Research in these countries tends to focus primarily on problems (i.e., negative health behaviors and poor health outcomes) at the individual level rather than on resilience (positive health behaviors and outcomes). Perhaps because of this tendency, most SGM-related research draws on the minority stress model. This theoretical perspective posits that sexual- and gender-related health disparities are largely caused by unique stressors (e.g., stigma, harassment) that SGM people experience, in addition to everyday stressors experienced by individuals in the general population. The Dutch studies reviewed in this project also mostly focused primarily on problems and gave very little attention to resilience. Further, we found no studies that directly assessed links between structural stigma and health outcomes. Attention was primarily given to individual-level stressors (e.g., stigma consciousness [vigilance regarding expectations of stigma and rejection], internalized stigma/homophobia [negative feelings about one’s own sexual or gender minority status]); or to interpersonal stigma [e.g., discrimination, bullying and other forms of violence and trauma]). Given findings of persistent health disparities—despite the high level of SGM supportive policies in the Netherlands—research is needed to better understand the different levels of stigma (structural, interpersonal, individual) and how they interact to impact health among SGM people [34].

A particularly important area for structural-level research not addressed in the studies we reviewed is healthcare. Healthcare providers’ cis/heteronormative biases, stereotyping, prejudice, and clinical uncertainty have been shown to contribute to health disparities in other countries [121–124]. To avoid discriminatory or stigmatizing treatment, SGM people often postpone healthcare or conceal their sexual or gender identity when seeking care [122], thereby reducing providers’ ability to understand and address their health needs. This is particularly the case for transgender people who report the greatest levels of dissatisfaction and reluctance to seek healthcare and medical treatment among all SGM population groups [125–128]. A growing body of evidence points to deficiencies in health professionals’ education as a major reason for physicians’ and nurses’ lack of knowledge, biases and stereotypes regarding SGM people and their health [129–134]. Recent studies evaluating SGM health content in medical and health professions curricula have found major gaps and unmet learning needs among both students and educators [134, 135], despite the international availability of educational frameworks and materials [136, 137]. Currently, most education on SGM health is informal and supported by organizations such as the ‘Alliantie Gezondheidszorg op Maat’ (an alliance of several non-governmental organizations that provide educational material for the health and care sector), Roze in Wit (Pink in White, a national organization of SGM physicians who push for change at the policy and institutional levels) and “Treat it Queer” (an advocacy group of young physicians who teach clinical workshops and develop educational materials) [138]. The lack of inclusion of SGM related information in health professional training is likely linked to limited research and information about the health of SGM population groups. Strategies to improve access to knowledgeable and sensitive healthcare are essential.

### Gaps in the literature

Findings from this review point to many gaps in SGM research on substance use and mental health in the Netherlands. For example, only one study focused exclusively on adult SMW; this study consisted of a qualitative exploration of coming out, sexuality, mental health, and discrimination among 10 lesbian and bisexual women with mild intellectual disabilities. More attention was given to sexual minority girls, but mostly in the context of examining sex/gender differences in mental health or substance use outcomes among SGM adolescents and young adults. The lack of attention to sexual minority girls and women is important given that research in other parts of the world consistently finds sex/gender differences in health outcomes. Indeed, several studies (e.g., Bos, van Beusekom [76], la Roi, Kretschmer [16]) in this review found such differences. There was some evidence that bisexual girls and women are at greater risk for substance use and poor mental health than lesbian girls and women, but more research is needed, both to replicate these findings and to understand what factors may contribute to this heightened risk. Moreover, as noted above, only two studies focused on older SGM adults, and few studies focused on the experiences of non-binary individuals.

In addition to the paucity of studies on SM girls and women, bisexual men, older SGM adults, and nonbinary people, nearly all study samples were quite homogenous; race or ethnicity was rarely reported. Exceptions include a study of gay Iranian men [80] and another of transgender Iranian people [108]. The growing emphasis on intersectionality in the literature from other countries highlights the importance of factors such as race/ethnicity, gender, socioeconomic status, and other marginalized statuses in studies of SGM health [139–144]. These factors may interact to mediate or to moderate the impact of stigma on health status differently across groups within the SGM population.

Although some studies had large sample sizes, primarily those that conducted secondary analyses of existing data sets, most samples were small and used non-probability sampling methods. Only 11 of the included studies (six of LGB adults, four of LGB youth, and one of TGD youth) used nationally representative probability samples. This limitation makes it difficult to compare the Netherlands with other countries and may contribute to over or under estimation of mental health and substance use disparities.

Additionally, many studies, especially those focused on youth, assessed sexual orientation based on sexual attraction. Studies of SGM adults sometimes used having a same-sex partner as a proxy for SGM status. Previous research has found that health concerns and health disparities vary based on how sexual minority status is assessed [145]. Further, as is the case in literature about transgender health more generally, terms referring to assigned sex at birth (“male” and “female”) and gender (“men” and “women”) in the Dutch studies we reviewed were sometimes used interchangeably. This contributes to confusion about whether health differences are due to sex, gender, both, or neither and complicates understanding of health disparities overall.

It was notable that most studies on substance use in the current review focused primarily on drug use during sexual activities among men who have sex with men and rarely used measures of substance use disorders. Although one study used alcohol and drug use disorder measures [106], these were used in association with changes during PrEP use. Studies that assess disparities in substance use disorders by sexual identity and gender identity using probability samples, or large nonprobability samples, are important to evaluate potential need for culturally appropriate treatment or other interventions designed to address hazardous alcohol or drug use.

Perhaps one reason for the somewhat narrow focus of SGM studies conducted in the Netherlands is the fact that a relatively small number of authors conducted many of the studies included in this review, particularly those with SGM youth. These authors work in departments and research groups that focus on youth and families more generally; to our knowledge, none of these departments or groups focus specifically on SGM youth.

### Limitations of our methods

Given the nature of scoping reviews—which aim to synthesize an existing and evolving body of literature to determine knowledge gaps and identify areas for future empirical work—we may have missed studies or other literature that could have provided a more complete understanding of SGM health in the Netherlands. In keeping with scoping review methodology, we did not address risk of bias or evaluate other limitations of individual studies. In addition, we made the decision to limit the time frame for included studies to 2010–2022. By doing so, we may have missed studies with useful findings that were published before 2010.

### Major recommendations

#### The Netherlands may need to examine its stance on equality

The paradox of persistent SGM health disparities despite the Netherlands’s strong history of supportive policies highlights the importance of recognizing that health inequalities have multiple root causes and that reducing these inequalities is complex. Ironically, the Netherlands’s strong reputation as a progressive and tolerant country, and its stance on equality, may inadvertently contribute to the problem. As noted by a United Nations Special Rapporteur on racism, racial discrimination, and related intolerance: “The paradox in the Netherlands is that insistence that equality and tolerance already exist actually operates as a barrier to achieving this equality and tolerance in fact.” Thus, “this insistence makes it difficult to mobilize the resources and action necessary to ensure equality, non-discrimination and inclusion for all” [146]. If the Dutch believe that the goal of creating a completely equal society has been achieved, explicit attention to the health and welfare of SGM people and other marginalized populations may be perceived as no longer necessary.

Further, drawing on Butler’s [147] theory of normalization and Goffman’s [148] theories of stigmatization, Robinson [149] conducted in-depth interviews with SGM people in the Netherlands to explore how the country’s social acceptance and legal protections impact their lives. Based on information gleaned from these interviews Robinson argues that the danger of acceptance is invisibility, shame, and fear for those who assimilate, and marginalization for those who do not conform to assimilationist discourses, including transgender individuals and others who do not conform to traditional gender roles or expression. Robinson concludes that new approaches to dismantling heteronormativity are necessary to achieve genuine acceptance for SGM people in the Netherlands.

#### Need for theoretical perspectives other than minority stress and for structured programs of research and research funding

Other than frequent use of the minority stress theoretical framework, studies included in this review had little in common. For example, research questions, definitions of measures, and outcomes were often quite disparate, making comparisons across study findings difficult, if not impossible. The almost exclusive reliance on the minority stress model is also a limitation. Models and frameworks that incorporate a broader range of social determinants of health, interpersonal relationships, and life course perspectives are needed to guide a more cohesive body of research about SGM health in the Netherlands. For example, Diamond and Alley [47] argue that the narrow focus on minority stress in SGM health research has likely obscured important information about factors underlying sexual- and gender-related health disparities. In particular, they assert that the lack of sufficient social safety is a primary cause of stigma-related health disparities and an important target for intervention. Social Safety refers to “social connection, social inclusion, social protection, social recognition, and social acceptance” (p. 5), which, based on limited findings in this review, appears to be a key factor contributing to heightened risk for substance use and poor mental health among SGM people living in the Netherlands. For example, a number of study findings highlighted the negative effects of rejection, social isolation and lack of social recognition on SGM adults’ mental health [81, 97] and on SGM youth’s mental health [16, 53, 57, 59, 71, 75]. The impact of structural factors (e.g., socioeconomic status, geography, healthcare contexts and experiences) are also important social and contextual determinants of SGM health outcomes that need to be better understood. According to Fundamental Cause Theory, health inequalities persist even when risk factors change over time because, for example, individuals who are part of lower-status groups have less access than those of higher-status groups to health promoting or protecting resources, such as knowledge, prestige, power, and supportive social connections [9]. Alternative theories such as Social Safety and Fundamental Cause Theory hold promise for deepening understanding of mechanisms underlying SGM health disparities and factors other than minority stress that contribute to poorer health among SGM people and may help explain the disconnect between the high level of SGM supportive laws and policies and persistent health disparities in the Netherlands. With this information, tailored interventions for SGM people of various ages, genders, and socioeconomic status can be developed. Longitudinal cohort studies using representative samples are the gold standard and could greatly facilitate understanding of the impact of historical changes and age-varying developmental factors.

Published reports of the studies included in this review rarely mentioned funding and we are aware of no funding sources specifically for SGM health research in the Netherlands. Lack of funding may reflect the lack of recognition among funding bodies that SGM people experience poorer health than heterosexual people. This, in turn, may help explain why sexual orientation and gender identity data have not been systematically assessed in national health and epidemiological surveys in the Netherlands—and contributes to the major gaps in knowledge observed in our review. Funding for and dissemination of research regarding antiretroviral treatments greatly reduced SGM disparities related to HIV/AIDS in the Netherlands [150], but other major health concerns among SGM people, such as those arising from stigma, trauma, stress, and violence, remain understudied.

To move toward a more comprehensive, health justice driven SGM research landscape it is important that individual researchers in the Netherlands (and Flanders) join forces [151]. Practically, this would mean active involvement in shaping research programs of the major funding bodies (e.g., NWO and ZonMW). In addition, it would entail a commitment to interdisciplinary and cross disciplinary research. Health research priorities in the Netherlands are linked to societal challenges defined by the Dutch government; these challenges inform how research funding is allocated. Recently, socioeconomic inequality has been prioritized as an important research and policy target. To maximize SGM research funding opportunities efforts might be framed to focus on understanding the interplay among socioeconomic status or class, SGM status, and health. The Social and Cultural Planning office has argued that target group approaches—such as research focused on SGM health—will have negligible impact because such groups include people of diverse socioeconomic backgrounds [151, 152]. Consequently, the current emphasis on class-based disparities and the ensuing need for national remediation strategies in which class relationships are explicitly recognized has implications for SGM research agendas. For instance, it implies that investigations into how socioeconomic status impacts the health of SGM subgroups—drawing on theoretical perspectives such as the Fundamental Cause Theory [153]—are more closely aligned with current research priorities. In addition, given the emphasis on community participation in research, SGM studies that incorporate these methods may be attractive to Dutch funding agencies (see, e.g., Ünsal, Demetrovics [151]).

## Conclusion

To address and eliminate SGM health disparities, greater understanding of the mechanisms underlying these disparities is essential. Our findings point to major gaps in the literature related to Dutch SGM people’s health regarding the sub-populations studied, the theoretical perspectives used, and the overall limited research on the topic. Findings also point to the need for resources that support collaborative SGM health focused research teams in the Netherlands and the need for researchers in the Netherlands and elsewhere to move beyond the current narrow focus on minority stress to understand causes of sexual and gender identity related health disparities. Remediating SGM health disparities in the Netherlands and in other countries requires a multifaceted approach that addresses the fundamental causes of inequalities, focuses on preventing harmful wider social influences, and works to mitigate the negative effects of inequalities on individuals.

## Data Availability

N/A.
